# *Acinetobacter baumannii* as a Model for the Study and Application of Gram‐Negative Outer Membrane Vesicles: A Systematic Review

**DOI:** 10.1111/1751-7915.70207

**Published:** 2025-09-04

**Authors:** Beatriz Cano‐Castaño, Mireia López‐Siles, Francesca Nonnoi, Astrid Pérez, Andrés Corral Lugo, Michael J. McConnell

**Affiliations:** ^1^ Intrahospital Infections Laboratory Instituto de Salud Carlos III (ISCIII), National Centre for Microbiology Madrid Spain; ^2^ Escuela Internacional de Doctorado, Ciencias de la Salud Universidad Nacional de Educación a Distancia (UNED) Madrid Spain; ^3^ Microbiology of Intestinal Diseases, Biology Department Universitat de Girona Girona Spain; ^4^ Protein Synthesis Quality Control Institute of Genetics and Development of Rennes Rennes Cedex France; ^5^ Department of Biological Sciences University of Notre Dame Notre Dame Indiana USA; ^6^ Eck Institute for Global Health, University of Notre Dame Notre Dame Indiana USA

**Keywords:** *Acinetobacter baumannii*, antibiotic resistance, biogenesis, biotechnology, OMVs, outer membrane vesicles, proteins, vaccine, virulence factors

## Abstract

Outer membrane vesicles (OMVs) are extracellular nanostructures released from Gram‐negative bacteria. *Acinetobacter baumannii* OMVs (AbOMVs) have been extensively studied and can thus be used as a model for understanding multiple aspects of OMV biology. In this systematic review, we comprehensively assess the relevant literature covering AbOMVs and present these studies in the context of OMV biology in general. An overview of current knowledge regarding AbOMV biogenesis is presented, focusing on the cellular, intrinsic and extracellular factors that induce AbOMV production. In addition, the components that form the AbOMVs, with an emphasis on protein content, are described. Different methodologies that have been used to isolate and purify the AbOMVs for different studies and applications are discussed. In addition, we provide a synthesis of the different roles played by AbOMVs in bacterial pathobiology, including the delivery of virulence factors, activation of immune response, gene transfer and antibiotic resistance. Finally, we detail the biotechnological applications that have employed AbOMVs, focusing on the development of AbOMV‐based vaccines. Altogether, this systematic review offers a perspective of current knowledge of AbOMV and serves as a case study of Gram‐negative OMVs in general.

## Graphical abstract

Investigation of outer membrane vesicles from *Acinetobacter baumannii* for potential biotechnological applications.

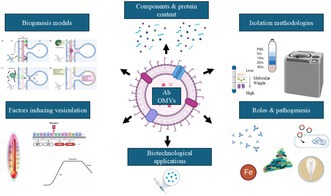

## 1 *Acinetobacter baumannii* As a Model System for Studying OMVs

*Acinetobacter baumannii* is an aerobic, non‐fermentative, oxidase‐negative and catalase‐positive opportunistic pathogen that causes community and nosocomial infections, predominantly ventilator‐associated pneumonia and bloodstream, urinary tract, skin and soft tissue infections (Sarshar et al. 2022). *A. baumannii* is a member of the antibiotic‐resistant ESKAPE pathogens, and is listed by the World Health Organization among the top priority pathogens in urgent need of the development of new antimicrobial therapies (López‐Siles et al. 2021; WHO Bacterial Priority Pathogens List 2024; CDC 2019). In recent decades, *A. baumannii* has emerged as a significant problem in intensive care units (ICUs) due to its intrinsic ability to persist in the hospital environment (Markogiannakis et al. 2008) and to resist antibiotic treatment, either through its extensive genomic plasticity or its ability to acquire genetic determinants of antibiotic resistance (Imperi et al. 2011; Olmeda‐López et al. 2021). In addition, its ability to evade the immune system (Morris et al. 2019) reduces the effectiveness of host defences. These capabilities have enabled multidrug‐resistant (MDR) strains of *A. baumannii* to cause ICU outbreaks worldwide, resulting in higher medical costs and increased mortality (Gramatniece et al. 2019).

**FIGURE 1:**
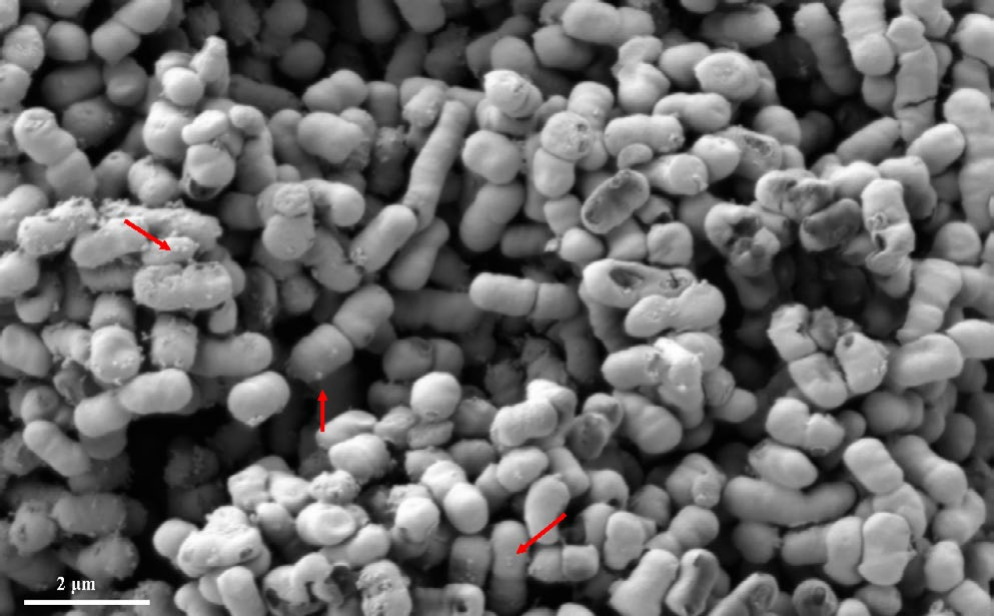
Visualisation of *Acinetobacter baumannii* ATCC 19606 producing OMV. Scanning electron microscopy of *A. baumannii* ATCC 19606 cells coated with a layer of gold. Red arrows indicate the position of AbOMVs around *A. baumannii* cells.

OMVs are 20–500 nm nanostructures produced and spontaneously released from the bacterial outer membrane (OM) (Toyofuku et al. 2023). They consist of spherical particles with an outer leaflet of lipopolysaccharide (LPS) and an inner leaflet consisting of phospholipid (Toyofuku et al. 2019). OMVs were first described using electron microscopy in 1960 in two genera of Gram‐negative bacteria: *Escherichia coli* and *Vibrio cholerae* (Work et al. 1966). Subsequently, OMVs have been identified in many Gram‐negative species, including *A. baumannii* (Figure 1) (Tiku et al. 2021) *Borrelia burgdorferi* (Skare et al. 1995), *Campylobacter jejuni* (Elmi et al. 2012), *Helicobacter pylori* (Windle 2021), *Klebsiella pneumoniae* (Lee et al. 2012), *Neisseria* sp. (Gerritzen et al. 2019), *Pseudomonas aeruginosa* (Wessel et al. 2013) and *Salmonella* sp. (Bai et al. 2014), among others.

In 1979, the first study describing AbOMVs was published, demonstrating how *Acinetobacter* sp. produced and encapsulated [3H] hexadecane using these nanostructures. AbOMVs were described as particles rich in phospholipids and LPSs, with a polypeptide composition similar to the OM of *Acinetobacter* sp. (Käppeli and Finnerty 1979). Approximately 20 years later, interest in studying AbOMVs was renewed due to the publication of an article detailing the isolation of AbOMVs from a clinical strain of *A. baumannii* . This study revealed the first proteome of AbOMVs and described their role in delivering virulence factors to host cells (Kwon et al. 2009).

The clinical relevance of *A. baumannii* , combined with its tractability, non‐fastidious growth, an expanding repertoire of genetic tools and animal models for studying pathogenesis, make *A. baumannii* a convenient system for studying OMV biogenesis and the role of OMVs in bacterial pathogenesis (McConnell et al. 2013). In this systematic review, we conducted a literature search in PubMed to identify studies on *A. baumannii* OMVs, including their biogenesis, protein content, factors that affects OMVs, described roles and future applications, from June 22, 2009, to July 8, 2025. The terms (‘ *Acinetobacter baumannii* ’ or ‘ *A. baumannii* ’) and ‘OMVs’ and (‘LPS’ or ‘LOS’) and ‘biogenesis’ and (‘proteins’ or ‘proteomic’) and ‘roles’ and ‘biotechnological applications’ were used to compile a total of 44 original research articles and 3 reviews, and full texts of the articles were reviewed.

## 2 AbOMV Biogenesis

Gram‐negative bacteria produce OMVs in vitro and in vivo, both during planktonic growth and biofilm formation, inside eukaryotic cells and within mammalian hosts during infection (Schwechheimer et al. 2013; Avila‐Calderón et al. 2021). The synthesis of a new OMVs begins with bulging of the OM and ends with the deposition of the OMV in the extracellular space (Schwechheimer et al. 2013). Evidence accumulated to date suggests that OMV biogenesis is a controlled process at the cellular level, rather than a stochastic event. During OMV biogenesis, the OM must be liberated from the underlying peptidoglycan (PG) through various covalent modifications. This allows the membrane to bulge outward, and with the assistance of certain proteins and lipids, the vesicle undergoes fission and detaches from the membrane (Schwechheimer et al. 2013). Although the onset of OMV formation may initially appear to be a damaging alteration of the OM, the beneficial functions that OMVs provide for the bacteria suggest that these alterations are part of a well‐structured physiological phenomenon. In these OMV models, it is believed that bacteria can use OMVs to improve their chances of survival and induce changes in their environment.

**FIGURE 2:**
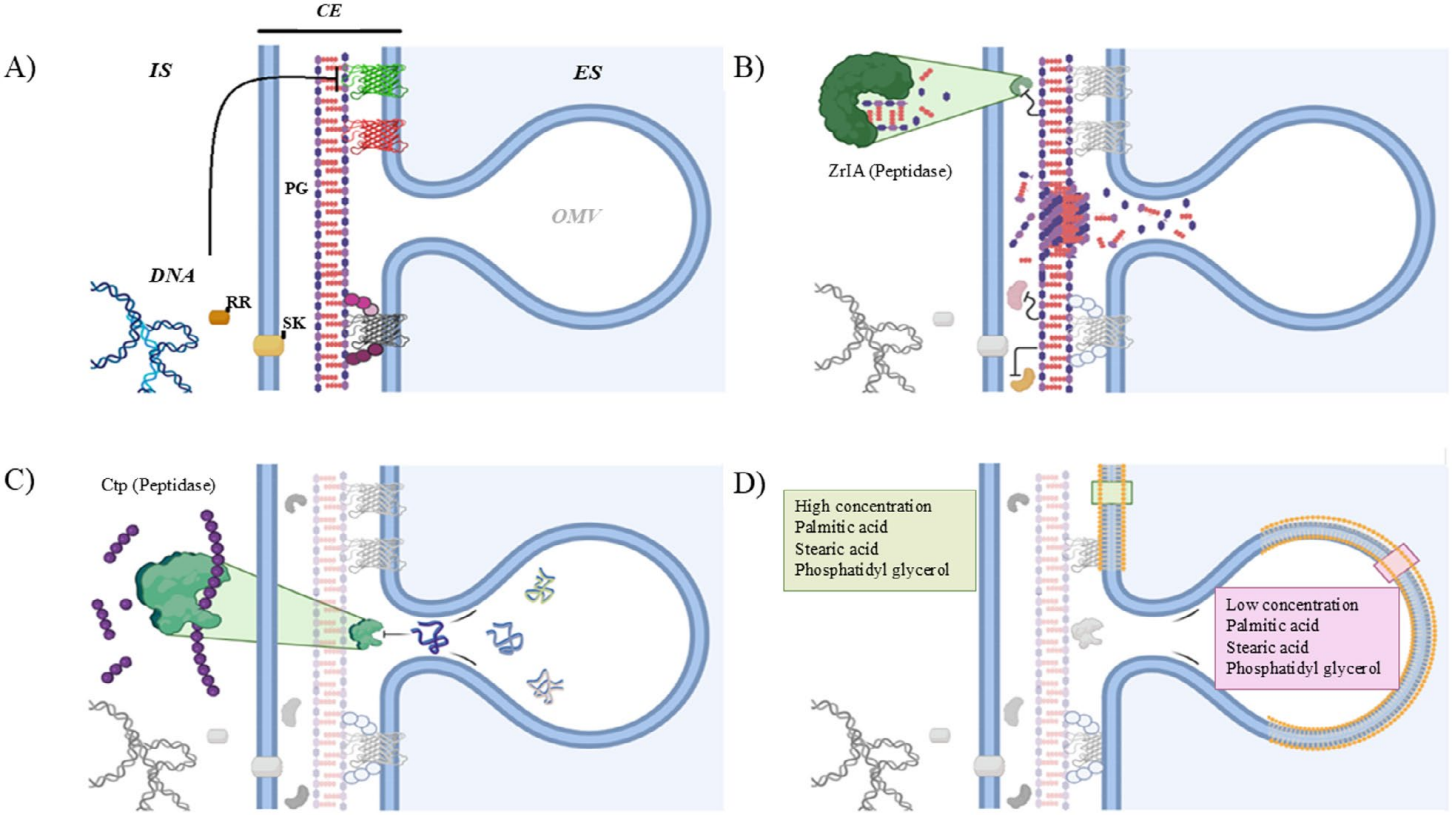
Biogenesis of AbOMVs based on four models. A schematic representation of the four models by which an OMV can be produced in *Acinetobacter baumannii* is shown. (A) In the OM and peptidoglycan (PG) cross‐linking model, AbOMV is formed as a consequence of the loss of the interaction between proteins of the outer membrane with the PG in the cellular envelope (CE), as is the case of the protein OmpA (green), OmpW red and the multiport complex that includes BamE (purple), which binds to peptidoglycan in several protein domains. The loss of these interactions facilitates AbOMV formation and placement in the extracellular space (SP). In addition, in the intracellular space (IS) the mutation in the sensor kinase (SK) of the two‐component system BfmS‐R affects the expression of *ompA* by the response regulator (RR) Bfms‐R, reducing its presence in the membrane and causing hypervesiculation phenotypes. (B) In the alteration in PG structure model, mutation in the zinc‐regulated peptidase ZrlA disrupts the PG recycling pathway, causing decreased membrane barrier function and aberrant PG muropeptide abundances. Therefore, accumulation of short fragments from PG reduces interactions between OM proteins and peptidoglycans, resulting in increased envelope permeability and hyperproduction of OMVs. (C) The model based on the accumulation of misfolded proteins is derived from CE stress response, and mutation in Ctp peptidase causes an accumulation of misfolded proteins, which exert pressure on the membrane, favouring the formation of the OMV. (D) The OM remodelling model, based on the lipid modification in certain areas of the CE, affects the composition of the lipids in the *A. baylyi* membrane which, in turn, affects the fluidity of the membrane, favouring the curvature of the vesicles.

To date, no specific biosynthetic pathway has been shown to be solely responsible for OMV production, nor has a specific signalling cascade been identified. However, work with mutant strains and bacterial cultures under different conditions provides clues that have resulted in different models for OMV biogenesis being proposed. There are currently four models that propose mechanisms for how bulge formation occurs (Figure 2). The first model of OMV biogenesis focuses on crosslinking interactions within the bacterial envelope, specifically among the OM, PG and inner membrane (IM). Proteins such as Lpp in *E. coli* create covalent crosslinks between the PG and OM, while other proteins like OmpA and Pal provide non‐covalent stability to the envelope structure (Schwechheimer et al. 2013). It is important to note that both the presence and the density of these interactions significantly influence OMV formation. For example, reducing the number of lipoproteins that are covalently anchored to the PG in certain regions of the *E. coli* membrane leads to localised changes, resulting in OM bulging and, consequently, OMV production (Oekstra et al. 1976). Other PG‐associated proteins shown to have a direct relationship with OMV biogenesis include OprI and OprF in *P. aeruginosa* , as mutants in these proteins produced 3‐ and 8‐fold more OMVs than the wild‐type (Wessel et al. 2013).

In *A. baumannii* , the C‐terminal region of OmpA has been shown to interact directly with the PG. Mutations in the *ompA* gene result in increased OMV production, probably because of decreased interactions between PG and OM, supporting this model (Moon et al. 2012). Additionally, mutations in the two‐component system sensor kinase *bfmS* decrease OmpA levels in the OM, leading to increased vesiculation. To date, *bfmS* is the only sensor identified in *A. baumannii* that regulates OMV production (Kim et al. 2019). Similarly, reduced expression of OM proteins OmpW and BamE weakens the linkage between the OM and PG, resulting in enhanced OMV release (Park et al. 2012).

The second biogenesis model proposes that it is the alteration of PG that initiates bulging, producing the OMV. Specifically, regions exhibiting elevated PG content, such as areas affected by mutations in PG recycling pathways, can produce protrusions, thereby triggering bulge formation and finally OMV generation (Koning et al. 2013; Schwechheimer and Kuehn 2015; Kim et al. 2021). A recent publication describing the Zinc uptake regulator (ZrlA) supports that the balance between PG synthesis and degradation may affect OMV production in *A. baumannii* (Figure 2B) (Kim et al. 2021). ZrlA is located in the inner membrane, where it displays peptidase activity and contributes to antimicrobial resistance and pathogenicity in *A. baumannii* . Hence, mutations in *zrlA* affect PG remodelling, leading to the accumulation of PG fragments and exhibiting a hypervesiculation phenotype. Further supporting evidence for this model is provided by the use of ceftazidime, which interferes with PG synthesis, resulting in the distortion of the PG layer and an increase in AbOMV formation at potential septum formation sites in ceftazidime‐treated bacteria (Koning et al. 2013).

In the third biogenesis model (Figure 2C), the accumulation of proteins in the periplasm causes membrane curvature, initiating membrane budding for OMV formation (Klimentová and Stulík 2015). Also, the accumulation of elongation factor EF‐Tu in the periplasmic space of *A. baumannii* promotes structural modification in OM, resulting in AbOMV secretion (Park et al. 2021). Protein analysis in *A. baumannii* showed that OmpA influences OMV protein composition, directly impacting OMV biogenesis (Moon et al. 2012). In addition, multiple cellular processes are controlled by the Ctp serine protease, including OMV biogenesis, PG remodelling and proteolytic cleavage of folded proteins in *A. baumannii* . Roy et al. postulated that the augmented vesiculation phenotype observed in the *ctp* knock‐out mutant results from the accumulation of misfolded proteins in the periplasm, consequent to the absence of the protease. This accumulation exerts increased pressure on the membrane, inducing membrane budding (Roy et al. 2021). Nonetheless, an alternative interpretation of this phenotype can be explained by the first model of biogenesis, as it affects the composition of PG and, therefore, the OM protein‐PG crosslink (Schwechheimer and Kuehn 2015; Avila‐Calderón et al. 2021).

In the fourth biogenesis model, membrane lipids play a key role in OMV biogenesis. OMVs harbour varying concentrations of certain fatty acids that dictate membrane flexibility. In both *P. aeruginosa* (Tashiro et al. 2011) and *Acinetobacter baylyi* (Fulsundar et al. 2014), it has been shown that high concentrations of long and unsaturated fatty acids, such as palmitic or stearic acid, together with the phospholipid phosphatidylglycerol, make the membrane more rigid, favouring the formation of membrane bulges. These fatty acids differ in concentration in the OM of the cell, and thus, this change in fatty acid composition also plays a role in OMV biogenesis (Figure 2D). In addition, temperature can affect the fluidity of lipids and, in consequence, that of the OM, leading to the release of OMVs. In some bacteria, low temperatures favour the formation of the bulge, probably because of the rigidity of the OM (McMahon et al. 2012). In contrast, high temperature induces OMV biogenesis in other species (McBroom and Kuehn 2007); this may be because high temperatures increase the fluidity of the lipids and, in consequence, that of the OM (Schwechheimer and Kuehn 2015). In *P. aeruginosa* , it has been reported that a change in temperature does not affect OMV production (MacDonald and Kuehn 2013).

LPS is a major component of the OM and plays a role in determining curvature and flexibility. OMV formation could be initiated by changes in LPS charge on the bacterial membrane. Studies show that *P. aeruginosa* strains with negatively charged LPS produce a higher quantity of OMVs compared to strains with neutral LPS. When LPS exhibits a heightened negative charge, it induces electrostatic repulsion within the OM, leading to OMV release (Li et al. 1996). Similarly, in *Salmonella enterica* serovar typhimurium, deacetylation of lipid A, which increases the negative charge of LPS, induces OMV production, further supporting the role of LPS in OMV biogenesis (Elhenawy et al. 2016). Specifically, in *A. baumannii* , the amount of lipooligosaccharide (LOS) in OMVs can be influenced by mutations in the enzymes responsible for lipid A synthesis within LOS, as well as by the overexpression of LOS synthesis enzymes such as the lipid A deacylase PagL (Badmasti et al. 2015). Interestingly, *A. baumannii* strains lacking LOS are still capable of producing OMVs (Pulido et al. 2020; Cano‐Castaño et al. 2024), indicating that LOS is not strictly necessary for OMV formation. In these LOS‐deficient strains, OMV production may proceed via alternative biogenesis pathways and could be induced as a stress response to the absence of LOS.

The available scientific evidence to date suggests that AbOMV biogenesis is a complex and multifactorial process that does not follow a single biogenesis model. Rather, OMVs are likely synthesised through all four different mechanisms depending on the physiological and environmental conditions in which *A. baumannii* is found, facilitating survival and modulating the surrounding environment.

## 3 Factors Modulating OMV Biogenesis in *Acinetobacter baumannii*

It is well known that the amount, size and protein content of OMVs differ depending on the strain and growth conditions, including environmental stressors, changes in growth medium and growth stage, together with the mutation of specific bacterial genes, highlighting the dynamic nature of OMV biogenesis under varying conditions (Table 1) (Fulsundar et al. 2014; Choi et al. 2014).

**TABLE 1:** Compilation of all factors that regulate OMV secretion in *Acinetobacter baumannii* .

	Effect	Strain	References
*Antibiotics and stress factors*			
Ceftazidime	Induce OMVs production by inhibition of peptidoglycan synthesis	*A. baumannii* AT	Koning et al. (2013)
Eravacycline	Cause OMVs production to protect cell from abiotic stress, increase the presence of stress‐related proteins and chaperones	*A. baumannii* ATCC 19606	Kesavan et al. (2020)
Gentamicin and Chloramphenicol	Changes in bacterial surface that results in increased OMVs production	*A. baylyi*	Fulsundar et al. (2014)
Imipenem	Promotes OMVs production and increase cytotoxicity, through the expression of carbapenemases and proteases	*A. baumannii* DU202	Yun et al. (2018)
D‐cycloserine	Peptidoglycan inhibitor that weakens *A. baumannii* cell wall integrity and stimulates OMVs production	*A. baumannii* DU202	Yun et al. (2018); Liao et al. (2015)
Pleural Fluid	Human serum albumin containing fluid that causes osmotic stress and DNA damage response and increases OMVs production	*A. baumannii* A 118	Martinez et al. (2019)
Cyprofloxacyn	Induces accumulation of damaged proteins and increases OMV release as a protective response	*P. aeruginosa*	Maredia et al. (2012)
Phage Lysin LysP53	Induces production of OMVs different in morphology, size and protein content (higher amount of cytoplasm proteins and lower cytotoxicity)	*A. baumannii* WHG40137	Li, Li, et al. (2024), Li, Xue, et al. (2024)
Peroxidase	Higher amount of proteins with oxidisable residues packaged into OMVs	*E. coli*	Orench‐Rivera and Kuehn (2021)
*Medium conditions*			
Temperature	Increased OMVs production at 37°C	*A. baylyi*	Fulsundar et al. (2014)
Desiccation	Increased OMVs production at 0.5 M of NaCl	*A. baylyi*	Fulsundar et al. (2014)
Nutrients stress condition	Increased OMVs production at 2.2 mM of succinate and 5.2 mM of glucose as carbon source; Expression of proteins related with different nutrients	*A. baylyi; P. putida*	Fulsundar et al. (2014), Choi et al. (2014)
UV2	Increased OMVs production at 20 min exposure	*A. baylyi*	Fulsundar et al. (2014)
*Growth stage*			
Early log phase	Low and small OMVs	*A. baumannii*	Koning et al. (2013), Huang et al. (2014), Huang et al. (2019)
Stationary growth phase	High and large OMVs	*A. baumannii*	Koning et al. (2013), Huang et al. (2014); Huang et al. (2019)
*Purification methodology*			
nOMVs	Larger OMVs	*A. baumannii* ATCC 17978	Li et al. (2020)
suOMVs	Higher amount of OMVs	*A. baumannii* ATCC17978; ATCC 19606	Li et al. (2020), Cano‐Castaño et al. (2024)
*Genes and elements*			
*hfq*	Plays a role in environmental adaptation and virulence modulating stress responses such as OMVs induction and decreases OmpA content in OMVs	*A. baumannii* ATCC 17978	Kuo et al. (2017)
*ABUW_1645*	Transcriptional regulator that increases OMVs production and protein content	*A. baumannii* AB 5075	Ahmad et al. (2019)
*BfmS*	Sensor kinase that negatively regulates the amount of OMVs	*A. baumannii*	Kim et al. (2019)
*blaOXA58*	Carbapenemase that is involved in OMVs production	*A. baumannii*	Liao et al. (2015)
*OmpA*	Role in integrity of bacterial surface; increased OmpA in OMVs increased toxicity in human cells	*A. baumannii* ATCC 19606	Skerniškytė et al. (2019)
*pmr operon*	Its upregulation is linked to overproduction of OMVs	*A. baumannii*	Park et al. (2021)
*EfTu*	It induces structural modification in OM resulting in OMVs secretion	*A. baumannii*	Park et al. (2021)
*ctp*	Serine protease, the knock out mutant is involved in vesiculation, induce larger OMVs and changes protein content	*A. baumannii* ATCC 17978	Roy et al. (2021)
*zrlA*	Zinc uptake‐regulator involved in bacterial morphology and in OMVs production	*A. baumannii* ATCC 17978	Kim et al. (2021)

Antibiotics and other stressors, particularly those targeting the bacterial envelope, have been shown to enhance OMV production and alter their protein composition in *A. baumannii* and other bacterial species. For instance, recent studies on *A. baylyi* demonstrated that even low‐level exposure to antibiotics like gentamicin and chloramphenicol leads to increased OMV production by inducing changes in bacterial surface architecture, creating more sites for vesiculation (Fulsundar et al. 2014). Similarly, imipenem, which disrupts cell wall synthesis by inhibiting β‐lactamase, resulted in a 2.2‐fold increase in AbOMV production. This was accompanied by an increase in cytotoxicity, notably through the expression of carbapenemases (OXA‐23, OXA‐58) and proteases (Yun et al. 2018; Liao et al. 2015).

Other antibiotics, such as D‐cycloserine and ceftazidime, which interfere with PG synthesis, also stimulate OMV production in *A. baumannii* (Koning et al. 2013). Recently, the effect of *Lippia macrophylla* essential oil together with ceftazidime has been associated with a higher production of AbOMVs (da Silva Cirino et al. 2024). Additionally, antibiotics like eravacycline have been shown to modify the protein profile of AbOMVs, increasing the presence of stress‐related proteins and chaperones critical for bacterial survival (Kesavan et al. 2020). In *P. aeruginosa* , ciprofloxacin‐induced DNA damage triggers the SOS response, leading to the accumulation of damaged proteins and an increase in OMV release as a protective response (Maredia et al. 2012).

In addition to antibiotics, AbOMV biogenesis and composition are influenced by culture conditions and environmental stressors. Studies on *A. baylyi* have shown that factors such as increased temperature, desiccation, nutrient deprivation and UV light induce changes in the bacterial surface and promote OMV release (Fulsundar et al. 2014). In various Gram‐negative bacteria, including *Pseudomonas* and *Acinetobacter* sp., these environmental factors lead to modifications in OMV protein content, particularly in MDR strains (Choi et al. 2014). Similarly, growth in nutrient‐rich media, such as LB, stimulates OMV production approximately two‐fold compared to minimal medium conditions (Fulsundar et al. 2014).

Environmental stressors also trigger significant changes in OMV. Studies using *A. baumannii* strains A118, A42 and AB5075 evidenced that exposure to pleural fluid, which induces osmotic stress, exhibited increased OMV release (Martinez et al. 2019).

A recent study employing the phage lysin LysP53 showed a higher production of OMVs different in morphology, size and protein content (higher amount of cytoplasm proteins) and that had lower cytotoxicity compared to natural secreted OMVs (Li, Li, et al. 2024; Li, Xue, et al. 2024). Similar to studies in *A. baumannii* , other research has shown that oxidative stress influences the protein composition of bacterial extracellular vesicles. A study on *E. coli* found that proteins with oxidisable residues were preferentially packaged into OMVs, highlighting how environmental stressors affect both the quantity and protein content of vesicles (Orench‐Rivera and Kuehn 2021).

Growth phase also directly affects the morphology and quantity of AbOMVs. During the early log phase, fewer and smaller OMVs (~30 nm) are produced, whereas a higher amount and larger OMVs (200–500 nm) are observed in the stationary growth phase (Koning et al. 2013; Huang et al. 2014, 2019). Interestingly, smaller OMVs tend to form at the distal ends of the bacteria, while larger OMVs are produced at the septa during bacterial division (Koning et al. 2013). *A. baumannii* MDR strains produce more OMVs than non‐MDR strains during the early stationary phase.

Numerous studies report the influence of different genes on the shape and production of AbOMVs (Kim et al. 2019; Skerniškytė et al. 2019), including transcriptional regulators, genes related to *A. baumannii* morphology and genes involved in antibiotic resistance. In the absence of *hfq*, a transcriptional regulator involved in stress responses like OM biogenesis, *A. baumannii* ATCC 17978 presents more difficulties in growth and manifests increased sensitivity to environmental stresses. The *hfq* mutant strain had reduced AbOMVs production and a lower amount of OmpA protein inside vesicles than the wild‐type strain, suggesting that *hfq* is necessary for OmpA expression and OMVs induction (Kuo et al. 2017). Overexpression of ABUW_1645 in *A. baumannii* resulted in higher protein concentration and number of OMVs per field imaged by microscopy (Ahmad et al. 2019). While some gene mutations involved in OMV biogenesis, such as *zrlA* (Kim et al. 2021) and *bfmS* (Kim et al. 2019), do not alter OMV size (~200 nm), mutations in the *ctp* gene result in larger OMVs, with an average size 26.9% larger than the wild‐type (Roy et al. 2021).

The method used for OMV purification significantly impacts both the size and quantity of the vesicles. Li et al. (2020) compared three different purification techniques and found that native OMVs extracted directly from bacterial cells were larger (average diameter of 269.9 nm) compared to those isolated from culture supernatants (average diameter of 142.9 nm) or through sucrose gradient extraction. Interestingly, sucrose gradient isolation yielded a higher quantity of OMVs (Cano‐Castaño et al. 2024). As recommended by the International Society for Extracellular Vesicles (ISEV) 2018, multiple centrifugation steps are required to obtain highly pure OMVs. However, as shown in Table S1, there is no single standardised method for OMV purification, nor a universally accepted quantification methodology. Ultracentrifugation is the most commonly used isolation technique, likely due to its speed, while both the bicinchoninic acid assay (BCA) and Bradford assay are frequently employed for quantification.

## 4 Protein Content of *A. baumannii* Outer Membrane Vesicles

**FIGURE 3:**
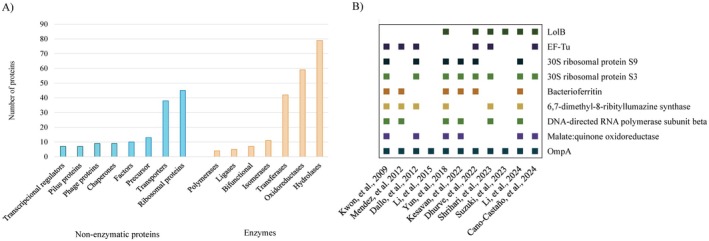
Comparative of proteomic studies of AbOMVs. (A) Enzymes and family proteins: The column chart represents different types of enzymes and protein families and their abundance in the 11 papers selected for the meta‐analysis. (B) Specific proteins: The squares in the chart show the presence of AbOMVs proteins in 11 different proteomic‐based publications: (1) Kwon et al. (2009). (2) Mendez et al. (2012). (3) Dallo et al. (2012). (4) Li et al. (2015). (5) Yun et al. (2018). (6) Kesavan et al. (2020). (7) Dhurve et al. (2022). (8) Shrihari et al. (2022). (9) Suzuki et al. (2023). (10) Li, Li, et al. (2024); Li, Xue, et al. (2024). (11) Cano‐Castaño et al. (2024). EF‐Tu, Elongation Factor Tu; RS, ribosomal protein.

Generally speaking, the composition of OMVs reflects the composition of the bacterial OM from which they are derived (Jan 2017). Among AbOMV components, most studies have focused on characterising OMV‐associated proteins. For the detection and identification of OMVs subproteomes, the most commonly used technique is liquid chromatography‐electrospray ionisation‐tandem mass spectrometry (LC–MS/MS). For this review, we identified 22 publications describing proteins associated with AbOMVs (Table S1), 11 of which provide a detailed list of proteins (Table S1). The proteins identified in these studies were used to determine which families and specific proteins have been found in AbOMVs (Figure 3).

Proteins in OMVs come from the cytoplasm, OM, inner membrane and the periplasm of the bacterial cell. Additionally, there are proteins whose origin remains unknown or that may come from multiple sites (Choi et al. 2014). Several authors report that the majority of proteins found in AbOMVs originate from the OM or an unknown location (Cano‐Castaño et al. 2024; Li et al. 2015). Conversely, other studies identify cytoplasmic proteins as the most abundant in AbOMVs (Suzuki et al. 2023). It is important to note that the method of purification used for isolating OMVs may affect the proteins identified in different studies.

In the studies included in this review, AbOMV proteins include those involved in biosynthesis and metabolism, such as DNA and RNA polymerases (Kwon et al. 2009), transferases like tyrosine‐protein kinase Ptk (Li, Li, et al. 2024; Li, Xue, et al. 2024) and different transglycosilases. Synthases and synthetases, such as ATP synthase subunit alpha and glutamine synthetase as well as decarboxylases, were also prominent (Kesavan et al. 2020). Additionally, enzymes with oxidoreductase activity were found, including malate:quinone oxidoreductase, quinoprotein glucose dehydrogenase, ubiquinol oxidase subunit 2, Cu/Zn superoxide dismutase (Dhurve et al. 2022), along with reductases, catalases, oxygenases and peroxidases. As shown in Figure 3A, the most abundant enzymes were hydrolases, which included proteases such as D‐alanyl‐D‐alanine carboxypeptidase (Li, Li, et al. 2024; Li, Xue, et al. 2024), as well as lipases, phosphatases, lyases and esterases. Other hydrolases related to antibiotic resistance were also identified, including 10 different types of b‐lactamases, such as class A (TEM), class C (AmpC) and class D (OXA‐24, OXA‐88, OXA‐133) (Cano‐Castaño et al. 2024; Li et al. 2015; Mendez et al. 2012). Less frequently identified enzymes included ligases, such as glutamate‐tRNA ligase and isomerases. Bifunctional proteins were also identified in AbOMVs (Li, Li, et al. 2024; Li, Xue, et al. 2024).

Multiple proteins with no predicted enzymatic activity have also been identified as components of AbOMVs (Figure 3B). OmpA (also referred to as Omp38) was detected in all studies and is one of the most abundant proteins in the *A baumannii* OM. It was found in both antibiotic‐susceptible strains, such as *A. baumannii* ATCC 19606 (Cano‐Castaño et al. 2024) and in MDR strains like A38 (Li et al. 2015), DU202 (Kwon et al. 2009), H120‐A2 (Mendez et al. 2012) and ATCC 19606 eravacycline resistant, as well as JU0126 (Kesavan et al. 2020). OmpA has also been reported in other antibiotic‐resistant strains, such as SA01 (Shahab et al. 2021) and in studies by Tiku et al. (2021). Moreover, OmpA is present in other species from the same genus, such as *Acinetobacter nosocomialis* (Nho et al. 2015) and *Acinetobacter radioresistens* (Fulsundar et al. 2015). In addition, OmpA homologues have been detected in OMVs from different Gram‐negative bacteria, including *Pseudomonas* (Choi et al. 2014).

Ribosomal proteins were the most diverse and frequently cited after OmpA. These included 30S and 50S ribosomal subunits. Several authors have described various types of transporters and ion channels, including carriers, ABC‐type transporters, RND multidrug efflux pumps (NodT family), TonB‐dependent receptors (Kesavan et al. 2020) and OMPs (such as OmpA, OmpW family proteins and Omp33‐36) (Rumbo et al. 2014). Additionally, essential proteins for iron storage, such as bacterioferritin, were found in 60% of the studies (Figure 3B).

Factors involved in replication and transcription, including regulators, have also been identified. Protein elongation factors (EF‐Tu, EF‐G) and other factors related to OM protein assembly, such as BamA, BamB, BamD and BamE, were found. OM lipoprotein precursors (OmpA, CarO, OmpW) are also consistently reported in most of the papers selected for analysis (Cano‐Castaño et al. 2024) as well as chaperones like Chaperonin GroEL or SurA (Dallo et al. 2012; Cano‐Castaño et al. 2024). Other less frequently mentioned proteins present in AbOMVs include pilus proteins, such as FilF (Mendez et al. 2012) and other proteins involved in adhesion, such as the two‐partner secretion system FhaB/FhaC, which are released via AbOMVs (Pérez et al. 2017). Additionally, phage proteins, including phage major capsid protein and Mu‐like prophage proteins, are mentioned in some studies, predominantly by Yun et al. (2018). Furthermore, proteins with toxic activity, such as RTX toxin‐related Ca^2+^‐binding protein, have also been identified (Li et al. 2015).

## 5 *Acinetobacter* *baumannii* OMVs and Pathogenesis

**FIGURE 4:**
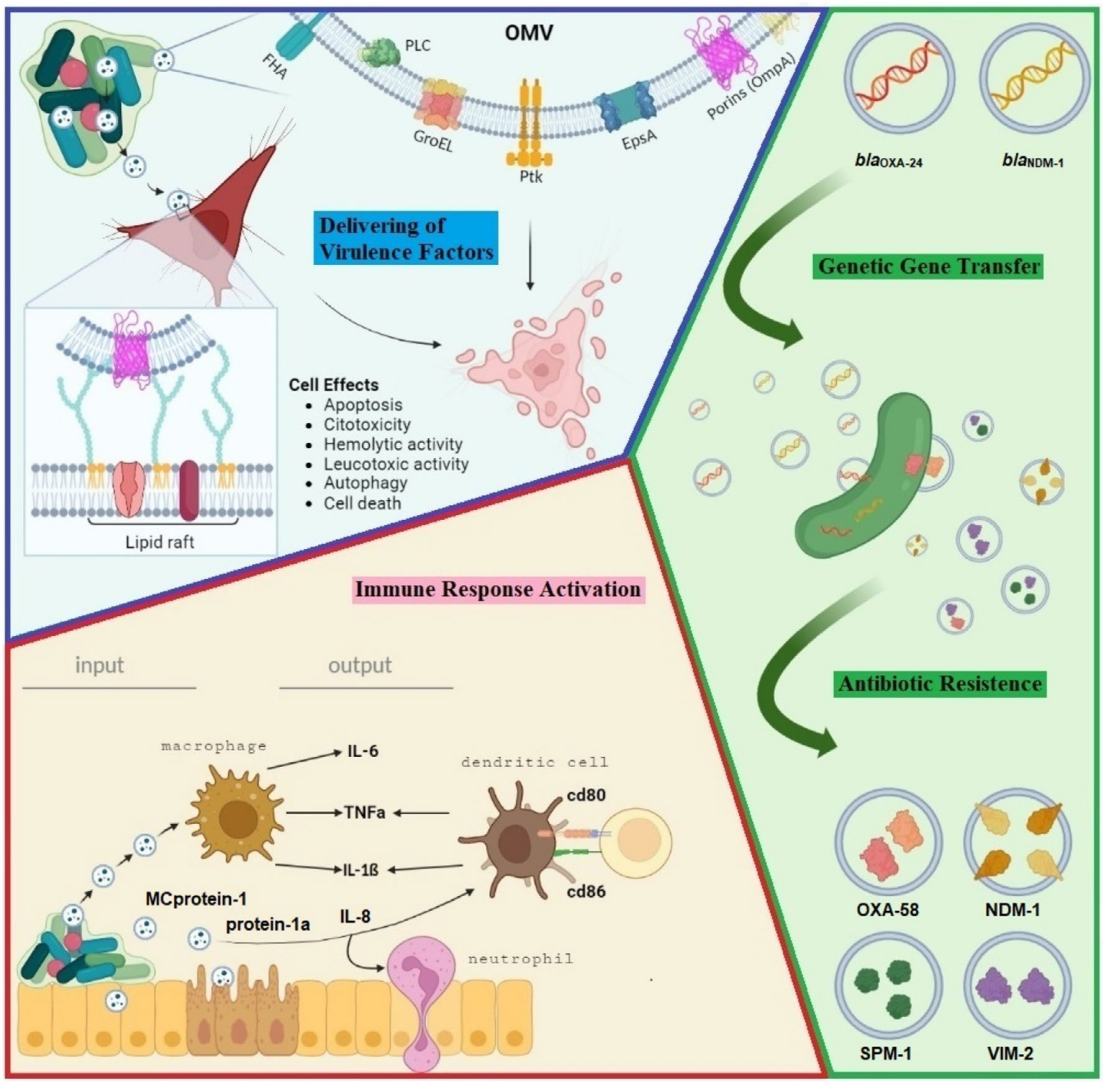
AbOMVs roles in multiple pathways. AbOMV secretion promotes *Acinetobacter baumannii* survival by delivering virulence factors (blue panel) and interacting with host cells during infection (pink panel) and horizontal gene transfer (green panel). Porins like OmpA, Omp33‐36, OmpW and Omp 38, along with proteins EpsA, Ptk, GroEL, PLC (phospholipase C) and FHA (hemagglutinin), among others, are secreted into the extracellular space of an *A. baumannii* biofilm via OMV. Newly secreted AbOMVs can interact with host cells through glycolipoprotein lipid microdomains called lipid rafts, causing cell‐damaging effects. AbOMV (input) interacts with host epithelium and activates the immune response via macrophages, neutrophils and dendritic cells, causing an inflammatory process (output) in host cells. In antibiotic resistance, AbOMVs play a role as carriers of resistance genes, either with linear or circular DNA (*bla*_OXA24_ and *bla*_NDM‐1_) that facilitate genetic transformation, and as antibiotic resistance proteins in cargo (OXA‐58 NMD‐1, SPM‐1 and VIM‐2) that degrade antibiotics in the environment, favouring the growth of antibiotic‐sensitive strains, including *A. baumannii* .

Although ongoing studies continue to elucidate the role played in bacterial physiology, OMVs have been shown to contribute specifically to four aspects of pathogenesis (Figure 4): (i) they facilitate bacterial survival under unfavourable conditions by enabling nutrient acquisition and promoting defense and resistance through the neutralisation of harmful agents and contributing to biofilm formation (Wang et al. 2015); (ii) they promote bacterial pathogenesis by shielding bacteria from the host immune response and containing virulence factors that destabilise the host cell (Rueter and Bielaszewska 2020); (iii) observations of vesiculation during infection indicate that OMVs serve as immunomodulatory agents and contribute to communication with the host cell (Ellis and Kuehn 2010; Kulp and Kuehn 2010; Schwechheimer and Kuehn 2015; Marion et al. 2019); (iv) OMVs have been associated with antimicrobial resistance through various mechanisms, including the release of antibiotic‐degrading enzymes, the sequestration of antimicrobials and the facilitation of resistance gene transfer (Fulsundar et al. 2014; Chatterjee et al. 2017; Kim et al. 2018).

Initial studies on the role of AbOMVs indicated their contribution to the delivery of virulence factors during infection. To date, the release of porins, EpsA, Ptk, GroEL, FilF, hemagglutinin like protein (FHA), tissue‐degrading enzymes and phospholipase C (PLC), among others, has been associated with AbOMVs (Jin et al. 2011; Rumbo et al. 2014; Li et al. 2015; Jha et al. 2017; Park et al. 2021). Given the involvement of some of these proteins in biofilm production, AbOMVs have been evidenced to promote enhanced biofilm formation (Park et al. 2021). In addition, it has been demonstrated that some of these effector molecules are more concentrated in AbOMVs than in the bacterial cell (Jha et al. 2017).

Upon release, OMVs interact with lipid rafts in the plasma membranes and deliver virulence factors to host cells (Jin et al. 2011). Hemolytic and leukotoxic activities (Jha et al. 2017), as well as host cell death and apoptosis in immune and connective cell tissue (Rumbo et al. 2014) and different cell types such as lung epithelium and macrophages (Skerniškytė et al. 2021), have been associated with AbOMVs. However, studies in *A. nosocomialis* reported differences in cytotoxicity depending on the cell line (Kim et al. 2016). On the one hand, OMV‐induced cell death in *A. baumannii* has been demonstrated to occur through the activation of caspases and modulation of autophagy, whose blocking enables this pathogen to persist intracellularly inside autophagosomes with the subsequent development of cytotoxicity (Rumbo et al. 2014). On the other hand, OMV‐induced apoptosis in *A. baumannii* has been associated with OmpA and the N‐terminal region of this protein (Jin et al. 2011). When taken up by mammalian cells, OMVs containing OmpA cause mitochondria fragmentation, producing an elevation in reactive oxygen species that leads to cell death, being OmpA proven to suffice to induce this cytotoxic effect (Tiku et al. 2021). Recently, AbOMVs have also been shown to activate the aryl hydrocarbon receptor (AHR) of host cells, impacting the host tryptophan metabolism and promoting AHR‐ and FOS‐mediated cytotoxicity of infected cells (Kew et al. 2024). Also, the small RNA (sRNA) content of OMVs interacts with targets in infected cells, thus contributing to the pathogenicity of the bacteria (Sarshar et al. 2022).

Furthermore, mutations in *ctp* and *trxA* induce changes in OMV protein content, exhibiting higher cytotoxicity (Roy et al. 2021; Shrihari et al. 2022). Additionally, OMV cytotoxicity can be regulated by OmpA released via OMVs. Kim et al. (2019) described how increasing OmpA expression in OMVs resulted in increased toxicity to human cells. In addition, a higher phospholipase C activity in AbOMVs released by clinical strains compared to the reference strain ATCC 19606 indicates greater virulence potential from MDR strains of *A. baumannii* (Jha et al. 2017).

Concerning cell interaction and immune response activation, it has been shown that the surface‐exposed membrane proteins in AbOMVs substantially contribute to the proinflammatory response in mouse macrophages (Skerniškytė et al. 2021). Additionally, the expression of proinflammatory cytokine genes, interleukin (IL)‐1ß and IL‐6, TNFα, chemokine genes, IL‐8, macrophage inflammatory protein‐1α and monocyte chemoattractant protein‐1, has been shown in epithelial cells exposed to AbOMVs in a dose‐dependent manner (Jun et al. 2013; Marion et al. 2019). Early inflammatory processes, such as vacuolisation, detachment of epithelial cells and neutrophilic infiltration, have been observed in mice injected with AbOMVs (Jun et al. 2013; Marion et al. 2019). These AbOMVs have been reported to induce a strong innate immune response (Li et al. 2015). An independent study also demonstrated that AbOMVs could activate bone marrow‐derived dendritic cells to promote Th2 activity together with the humoral immune response (Cai et al. 2019). Studies in TLR2‐ and TLR4‐deficient mice elucidated that the proinflammatory response was partly inhibited, thus highlighting the importance of TLR in AbOMV‐induced inflammation (Marion et al. 2019). Altogether, these studies point out that AbOMVs mediate systemic inflammation during infection and play an important role in activating both innate and adaptive immune responses.

AbOMVs have been shown to play a role in antibiotic resistance, specifically carbapenemases. NDM‐1, VIM‐2 and SPM‐1 have been found in AbOMVs (López et al. 2019). Studies based on OXA‐58 have demonstrated the selective release of this enzyme via AbOMVs after a Sec‐dependent periplasmic translocation. In addition, this study evidenced a different biological role of OXA‐58 secreted via AbOMV from that of AbOMV‐independent secretion (Liao et al. 2015). A recent study demonstrated that OMVs from a polymyxin B‐resistant *A. baumannii* protected susceptible strains of this species and also members of the human gut microbiome against polymyxin B treatment (Park et al. 2021). Thus, AbOMVs can prepare the niche for other microorganisms. On the other hand, it has been reported that *Acinetobacter* spp. can benefit from OMVs released by other microorganisms to resist antibiotics. For example, OMVs from *E. coli* MG1655 protect against membrane‐active antibiotics colistin and melittin but not against ciprofloxacin, streptomycin and trimethoprim (Kulkarni et al. 2015). In line with this, *Moraxella catarrhalis* OMVs protected *A. baumannii* against membrane‐targeting agents (Roszkowiak et al. 2019).

Another role of OMVs associated with antimicrobial resistance is the transfer of resistance determinants and contribution to horizontal gene transfer. The first evidence of this function was elucidated through the plasmid‐borne *bla*_OXA‐24_ gene. Co‐incubation of OMVs obtained from resistant clinical strains of *A. baumannii* conferred full resistance to carbapenems to the susceptible *A. baumannii* ATCC 17978. New AbOMVs harbouring this gene were released by this strain after being transformed with the original OMV‐mediated plasmid (Rumbo et al. 2011). The presence of *bla*_NDM‐1_ and *acc*(*6′*)*‐Ib‐cr* genes from an *A. baumannii* strain of ST 1462 was also transferred via OMVs to both *A. baumannii* ATCC 19606 and *E. coli* . This was further phenotypically confirmed by elevated MIC values to ß‐lactams (Chatterjee et al. 2017). These studies have also established that the transformation frequency in different *Acinetobacter* spp. ranges between 10^−5^–10^−8^, with transfer efficiencies of approximately 10^3^ and 10^2^ per μg of vesicular DNA, and that AbOMVs interact with recipient cells in different ways depending on the recipient species (Fulsundar et al. 2014; Chatterjee et al. 2017). Similarly, it has been shown how AbOMVs can transport other plasmids and phage genomes across mammalian tissues and cells (Dhurve et al. 2024).

Regarding nutrient acquisition, AbOMVs have been specifically reported to play a role in iron uptake (Dhurve et al. 2022) as well as in *P. aeruginosa* (Lin et al. 2017). OMVs carry proteins involved in metal degradation, which may enable them to sequester and concentrate metals around the bacterium, making these nutrients more accessible for absorption and subsequent use (Lee et al. 2008).

## 6 *Acinetobacter baumannii* OMVs as Vaccines

OMVs are a well‐established platform for vaccine development, as they naturally present bacterial antigens capable of inducing strong immune responses. To date, two licensed vaccines incorporate OMVs in their formulation: the meningococcal group B vaccines Bexsero (developed by Novartis, Research C for BE and BEXSERO 2023) and VA‐MENGOC‐BC. OMVs from other pathogens, such as *A. baumannii* and *Shigella*, are also being explored as next‐generation vaccine platforms (McConnell et al. 2011; Qasim et al. 2022). In addition, in PubMed, there are more than 400 papers on this topic (last accession: October 2024), with over 50% published in the last 10 years, which reflects the growing interest in OMVs as a promising and versatile platform for vaccine development. Given the growing need for vaccines against antibiotic‐resistant Gram‐negative infections, the use of OMVs in the development of novel immunisation approaches may be especially clinically relevant (García‐Quintanilla et al. 2013, 2016; Li et al. 2022; Ye et al. 2023).

In addition to clinical evaluation of licensed OMV‐based vaccines, such as Bexsero, multiples studies have evaluated the safety profile of OMVs as vaccine platform, reporting an overall acceptable reactogenicity with mild and transient side effects such as pain, erythema or low‐grade fever (Gorringe et al. 2009; Nøkleby et al. 2007; Holst et al. 2013). Preclinical studies using OMVs from various Gram‐negative bacteria, including *N. meningitidis, A. baumannii* and *Shigella*, have shown that toxicity can be reduced through genetic modifications, such as detoxifying LPS or removing Lipid A (Moffatt et al. 2010; Badmasti et al. 2015).

Given that inactivated whole cells from different bacteria, including *A. baumannii* , have been used as vaccines to elicit antibodies against the bacterial antigens and provide protection in animal models (McConnell et al. 2011), and considering that OMVs contain components similar to the bacterial OM (Pulido et al. 2020; Avila‐Calderón et al. 2021), one of the main biotechnological applications of OMVs is their use as antigens to generate OMV‐based nanovaccines against MDR *A. baumannii* (McConnell et al. 2011; López‐Siles et al. 2021; Pulido et al. 2020; Cano‐Castaño et al. 2024).

AbOMVs derived from laboratory and clinical isolates have been shown to stimulate the immune response, increasing levels of IgG, IgG1, IgG2c, IgM, IL‐6 and IL‐1β, while reducing cytokine accumulation in fluids (Li, Li, et al. 2024; Li, Xue, et al. 2024). Additionally, vaccination with AbOMVs protects mice from infection with clinical *A. baumannii* isolates, as demonstrated in pneumonia and sepsis models. Vaccinated mice showed reduced bacterial load in organs and fluids (bronchoalveolar lavage fluid, lungs and spleen) compared to nonimmunised controls (Huang et al. 2014; Jun et al. 2013; Higham et al. 2023). Furthermore, serum from AbOMV‐immunised mice promotes opsonophagocytic killing of *A. baumannii* , and passive immunisation has been shown to induce IgG and IgA production (Huang et al. 2014; Li et al. 2020; Pulido et al. 2020; López‐Siles et al. 2021; Bjanes et al. 2023).

Not only have native AbOMVs been proven to be highly immunogenic and induced an immune response against *A. baumannii* infection in animal models but also a study published in 2016 engineered OMVs with the proposal to obtain an antigen delivery platform (Huang et al. 2016). They used the well‐known OM protein Omp22 from *A. baumannii* and the *E. coli* OMV to produce AbOmp22‐OMVs by recombinant gene technology. Immunological analysis using AbOmp22‐OMVs in a murine sepsis model showed a similar response observed in previously conducted analysis using AbOMV in active and passive immunisation, demonstrating that OMVs can express heterologous antigens to protect against several pathogens (Huang et al. 2016).

Additionally, reducing the toxicity of AbOMVs LOS is an important objective in the construction of OMV‐based nanovaccines, providing a benefit in the use of OMVs in clinical treatments or prophylaxis due to the cytotoxicity produced in mammals by LOS, LPS and endotoxin (Daneshian et al. 2006). Mutations in the *lpxA*, *lpxC* and *lpxD* genes involved in lipid A biosynthesis cause a complete loss of LOS in *A. baumannii* (Moffatt et al. 2010), while mutations in the two‐component system PmrA/PmrB induce modifications in LOS (Lesho et al. 2013). Due to total loss of LOS, *A. baumannii* produces free LOS OMVs (Pulido et al. 2020). These AbOMVs, isolated from an *A. baumannii* ∆*lpxD* mutant, were used as a nanovaccine candidate. Similar to AbOMVs with LOS, AbOMVs without LOS induced an antibody response (IgG, IgM, IgG1 and IgG2c) and reduced bacterial load; however, the absence of LOS reduced the protection of the mice. Nonetheless, the authors have demonstrated that this issue can be resolved by increasing the concentration of the AbOMVs 10‐fold during immunisation (Pulido et al. 2020).

Another vaccine candidate was developed from a strain of *A. baumannii* that overexpressed 3‐O‐deacylase PagL, an enzyme that reduced the endotoxicity of LOS (Badmasti et al. 2015). Immunological studies have demonstrated that this candidate robustly induces humoral and cellular immune responses, providing complete protection against *A. baumannii* infection. Considering that mice immunised with AbOMVs produce sera with high immunoglobulin content, and that they were used in passive immunisation with promising results, more advanced studies have proposed the use of combination therapy. This involves using these sera together with quinolone antibiotics, which helps to reduce bacterial loads in the lung and spleen in mice compared to monotherapy. The rationale behind this strategy is that anti‐AbOMV antibodies primarily enhance the intracellular accumulation of antibiotics by affecting porins, thus improving susceptibility to quinolone antibiotics (Huang et al. 2019).

Different administration routes for AbOMV vaccines have been tested to optimise protective efficacy. Intramuscular immunisation has demonstrated strong protection in mouse models of both pneumonia and bacteremia. In contrast, intranasal immunisation has shown good protection against bacteremia but was less effective in pneumonia models (Li, Li, et al. 2024; Li, Xue, et al. 2024). Notably, some studies suggest that intranasal immunisation is the most effective for reducing upper airway colonisation compared to intramuscular (Li et al. 2020) and subcutaneous administration (Higham et al. 2023). These findings highlight the importance of selecting the appropriate route of administration to maximise immune protection against different infection types.

In addition, new technologies are being employed with OMVs, as demonstrated by Bjanes et al. (2023), where gold nanoparticle cores coated with AbOMVs induced robust IgG titers in rabbits. This approach represents an innovative step in enhancing vaccine efficacy using OMVs.

## 7 Concluding Remarks

As described in the sections above, AbOMVs represent a multifaceted aspect of bacterial biology, offering insights into bacterial survival mechanisms, virulence and resistance. Their biogenesis, regulated by protein interactions, PG dynamics and lipid composition, highlights their importance in adapting to environmental and antibiotic stress. Furthermore, their protein content plays a crucial role in antibiotic resistance and immune modulation, establishing them as key players in bacterial pathogenesis. The ongoing research into OMV‐based therapeutic applications, including vaccines and drug delivery systems, presents promising solutions to combat multidrug‐resistant infections. Ultimately, the continued exploration and engineering of OMVs have the potential to revolutionise modern medicine, offering novel avenues for treatment and prevention of infectious diseases.

## Author Contributions

**Beatriz Cano‐Castaño:** methodology, formal analysis, writing – review and editing. **Mireia López‐Siles:** methodology, writing – review and editing, validation. **Francesca Nonnoi:** methodology, validation, writing – review and editing. **Astrid Pérez:** supervision. **Andrés Corral Lugo:** conceptualization, writing – original draft, validation, visualization, writing – review and editing, project administration, supervision. **Michael J. McConnell:** funding acquisition, validation, writing – review and editing, formal analysis, resources.

## Conflicts of Interest

The authors declare no conflicts of interest.

## Supporting information


**Table S1:** mbt270207‐sup‐0001‐TableS1.docx.

## Data Availability

Data sharing not applicable to this article as no datasets were generated or analysed during the current study.
