# Supplementary material for: Acinetobacter baumannii as a Model for the Study and Application of Gram‐Negative Outer Membrane Vesicles: A Systematic Review
Source: Microb Biotechnol. 2025 Sep 4;18(9):e70207. doi: 10.1111/1751-7915.70207 (PMC12409660; doi:10.1111/1751-7915.70207)
Supplement: Supplementary file 1 — Table S1: mbt270207‐sup‐0001‐TableS1.docx. [file MBT2-18-e70207-s001.docx]

# Supplemental material of:

***Acinetobacter baumannii* as a Model for the Study and Application of Gram-negative Outer Membrane Vesicles: A Systematic Review**

# Authors

Beatriz Cano-Castaño, Mireia López-Siles, Francesca Nonnoi, Astrid Pérez, Andrés Corral-Lugo, Michael J. McConnell

# Supp Table 1. Compilation of studies related to *A. baumannii* OMV uses and production.

| **OMVs' application** | **OMVs isolation methodology** | **Strain** | **OMVs' size (nm)** | **OMV quantification** | **Paper** |
| --- | --- | --- | --- | --- | --- |
| Immunotherapeutic treatments against *A. baumannii* infections | Ultracentrifugation 100,000 x g, 2 h, 4 ͦC | 54 strains | - | Bicinchoninic Acid (BCA) Protein Assay | [Ahmad et al. (2019)](https://doi.org/10.1371/journal.pone.0210082) |
| *A. baumannii* pathogenesis | Ultracentrifugation with 40% sucrose, 150,000 x g | A118, A42 and AB5075 | - | BCA protein assay | [Martinez, J., Fernandez, J.S., Liu, C. et al. (2019)](https://doi.org/10.1038/s41598-019-53847-2) |
| *A. baumannii* pathogenesis | Ultracentrifugation 130,000 × g, 3 h, 4 ͦC | Ab169 | - | Bradford assay | [Skerniškytė et al. (2019)](https://doi.org/10.3390/molecules24101972) |
| *A. baumannii* pathogenesis | Ultracentrifugation 60,000 x g, 4 h, 4 ͦC | ATCC 19606 | - | - | [Badmasti et al. (2015)](https://doi.org/10.1159/000371815) |
| *A. baumannii* pathogenesis | Ultracentrifugation 100,000 x g, 2 h, 4 ͦC | ATCC 17978, Δ*Hfq* and *Hfq*C | - | - | [Kuo et al. (2017)](https://doi.org/10.3389/fmicb.2017.02068) |
| *A. baumannii* pathogenesis | Ultracentrifugation 200.000 × g, 90 min (2x) | AbH12O-A2 |  | Bio-Rad protein assay | [Pérez et al. (2017)](https://doi.org/10.1080/21505594.2016.1262313) *PS* |
| Gram negative pathogenicity (study) | Sucrose 50% ultracentrifugation 150,000 x g, 1 h, 4 ͦC | ATCC 17978 | - | BCA protein assay | [López et al. (2019)](https://doi.org/10.1038/s41467-019-11615-w) |
| *A. baumannii* pathogenesis | Ultracentrifugation 150,000 x g, 3 h, 4 ͦC | ATCC 17978 | 197,8 ± 16,0 | Modified BCA protein assay | [Kim, N., Kim, H.J., Oh, M.H. et al. (2021)](https://doi.org/10.1186/s12866-020-02083-0) |
|  |  | OH743 | 180,9 ± 25,9 |  |  |
|  |  | OH810 | 190,7 ± 18,2 |  |  |
| *A. baumannii* pathogenesis | Ultracentrifugation 50,000 × g, 3 h, 4 ͦC | ATCC 17978 | 28 - 130 | BCA protein assay | [Roy et al. (2021)](https://doi.org/10.3390/microorganisms9061336) *PS* |
|  |  | MR14 and MR14C | 47 - 200 |  |  |
| *A. baumannii* pathogenesis | Ultracentrifugation 150,000 x g, 3 h, 4 ͦC (x2) | ATCC 17978 | 193,7 ± 11,9 | Modified BCA protein assay | [Kim, S.Y., Kim, M.H., Kim, S.I. et al. (2019)](https://doi.org/10.1186/s12866-019-1679-0) |
|  |  | Δ*bfmS* | 186,8 ± 1,6 |  |  |
|  |  | *bfmSC* | 74,8 ± 1,3 |  |  |
| *A. baumannii* pathogenesis | Ultracentrifugation 100,000 x g, 12 h, 4 ͦC | ATCC 19606 | - | - | [Dallo, Shatha F et al. (2012)](https://doi.org/10.1100/2012/128705) *PSM* |
| *A. baumannii* pathogenesis | Ultracentrifugation 200,000 x g, 90 min, 4 ͦC | ATCC 17978; ATCC Δ*mapA* | - | Modified Bradford assay | [Rumbo et al. (2014)](https://doi.org/10.1128/IAI.02034-14) *PS* |
| *A. baumannii* pathogenesis | Ultracentrifugation 150,000 x g, 3 h, 4 ͦC | ATCC 17978 | < 100 | Modified BCA protein assay | [Moon DC, Choi CH, Lee SM, Lee JH, Kim SI, et al. (2012)](https://doi.org/10.1371/journal.pone.0038974) *PS* |
| Molecular biology (subproteome analysis) | Ultracentrifugation 150,000 x g, 2 h, 4 ͦC + sucrose gradient + ultracentrifugation 150,000 x g, 5 h, 4 ͦC | DS002 | 20 - 300 |  | [Dhurve et al., 2022](https://journals.asm.org/doi/10.1128/spectrum.00293-22) *PSM* |
| Molecular biology (subproteome analysis) | Same as [Dhurve et al., 2022](https://journals.asm.org/doi/10.1128/spectrum.00293-22) | DS002 | - | - | [Dhurve et al., 2024](https://journals.asm.org/doi/10.1128/spectrum.00817-24) *PSM* |
| MV characterization and pathogenesis | Centrifugation 40,000 x g, 1 h, 4 ͦC x2 | AB41 | O-IMVs: 125 - 160 | TEM | [Pérez-Cruz et al. (2015)](https://doi.org/10.1371/journal.pone.0116896) |
|  |  |  | OMVs: 20 - 40 |  |  |
| OmpA biotechology applications | Ultracentrifugation 100,000 x g, 1 h, 4 ͦC | SA01 | 50 | BCA protein assay | [Shahryari Shahab et al. (2021)](https://doi.org/10.1128/mSystems.01175-20) *PS* |
| OMVs biogenesis | Ultracentrifugation 150,000 × g, 3 h, 4 ͦC | ATCC 19606T, ΔAb*OmpA* | - | Modified BCA protein assay | [Moon, D.C., Choi, C.H., Lee, J.H. et al. (2012)](https://doi.org/10.1007/s12275-012-1589-4) |
| OMVs pathogenicity (subproteome analysis) | Ultracentrifugation 150,000 × g, 3 h, 4 ͦC + Optiprep density gradient 200,000 × g, 20 h, 4 ͦC | DU202 | - | Modified BCA protein assay; 0,3 μg/mL | [Yun, S.H., Park, E.C., Lee, SY. et al. (2018)](https://doi.org/10.1186/s12014-018-9204-2) *PSM* |
| OMVs pathogenesis | Ultracentrifugation 100,000 × g, 3 hr, 4 ͦC | ATCC 17978 --> PMR High strains | - | Bradford assay | [Park et al. (2021)](https://doi.org/10.7554/eLife.66988) *PS* |
| OMVs pathogenesis (extracelullar proteome analysis study) | Ultracentrifugation 100,000 × g O/N, 4 °C | ATCC 19606 | - | Bradford assay | [Roszkowiak et al. (2019)](https://doi.org/10.3390/ijms20225577) |
| OMVs immunostimulation | Ultracentrifugation 150,000 x g, 3h, 4 °C | ATCC 19606 | 40 - 70 | Modified BCA protein assay | [Jun SH, Lee JH, Kim BR, Kim SI, Park TI, et al. (2013)](https://doi.org/10.1371/journal.pone.0071751) |
| OMVs immunostimulation | Ultracentrifugation 130,000 x g, 3 h, 4 °C | Ab169 | - | Bradford assay | [Skerniškytė et al. (2021)](https://doi.org/10.3390/pathogens10040407) |
| OMVs immunostimulation | Ultracentrifugation 150,000 × g, 3 h, 4 °C + Optiprep sucrose gradient 200,000 × g, 2 h, 4 °C | ATCC 15150 | 30 - 100 | Bradford assay | [Marrion et al. (2019)](https://doi.org/10.1128/IAI.00243-19) |
| OMVs biogenesis and morphology | Centrifugation 3,000 x g, 10 min, 4 °C + centrifugation 17,000 x g, 1 h, 4 °C | ATCC 19606 | 30 - 80 & 200 - 500 | - | [Koning et al. (2013)](https://doi.org/10.1016/j.resmic.2013.02.007) |
| OMVs pathogenesis | Ultracentrifugation 150,000 x g, 3h, 4 °C | ATCC 19606; | < 100 | Modified BCA protein assay | [Jin JS, Kwon SO, Moon DC, Gurung M, Lee JH, et al. (2011)](https://doi.org/10.1371/journal.pone.0017027) *PS* |
| OMVs pathogenesis | Ultracentrifugaation 369548.3 g, 1 h, 4 °C | ATCC 19606; Δ*LpxA* | - | BCA protein assay | [Kew et al., 2024](https://doi.org/10.1038/s41467-024-52118-7) |
| OMVs pathogenesis (proteome analysis) | Ultracentrifugation 128,400 ×g, 4˚C, 90 min + Optiprep gradient 100,300 x g, 16 h, 4 °C + Ultracentrifugation 99,800 ×g, 4˚C, 3 h | ATCC 19606 | 200 | Bradford assay | [Suzuki et al., 2023](https://doi.org/10.1371/%20journal.pone.0283109) *PSM* |
| OMVs pathogenesis (proteome analysis) | Ultracentrifugation 150,000 x g, 2 h | KOtrxA | 20 - 95 | - | [Shrihari et al., 2022 *PSM*](https://pubmed.ncbi.nlm.nih.gov/34713732/) |
|  |  | Ci79 | 38 - 80 |  |  |
| OMVs pathogenesis (transmission of resistance genes) | Ultracentrifugation 200,000 x g, 2 h, 4 °C + ultracentrifugation 100,000 x g, 45 min, 4 °C + Optiprep density gradient 50,000 x g, 3 h, 4 °C | A_115, ST 1462 | 25 - 100 | Bradford assay | [Chatterjee et al. (2017)](https://doi.org/10.1093/jac/dkx131) |
| OMVs pathogenesis (transmission of resistance genes) | Ultracentrifugation 200,000 x g, 90 min, 4 °C | AbH12O-A2, AbH12O-CU3 and ATCC 17978 | 20 - 100 | Bradford assay | [Rumbo et al., 2011](https://doi.org/10.1128/AAC.00929-10) |
| OMVs pathogenesis (virulence study) | Ultracentrifugation 150,000 x g, 3 h, 4 °C | MDR A38, 5806 | 30 - 140 | Modified BCA protein assay | [Li et al. (2015)](https://doi.org/10.1016/j.micpath.2015.03.009) *PSM* |
| OMVs pathogenicity | Ultracentrifugation 150,000 x g, 3h, 4 °C | ATCC 19606T, | 80 ± 20 | Modified BCA protein assay | [Jha et al. (2017)](https://doi.org/10.1016/j.micpath.2017.08.048) *PS* |
| OMVs pathogenicity | Ultracentrifugation 40,000 r.p.m., 2 h, 4 °C | Ab19606, Ab17978 and Ab5075 | - | BCA protein assay | [Tiku, V., Kofoed, E.M., Yan, D. et al. (2021)](https://doi.org/10.1038/s41598-020-79966-9) *PS* |
| OMVs pathogenicity (antibiotic resistance) | Ultracentrifugation 100,000 x g, 1 h, 4 °C | Ec1003; Ab290 | 50 - 100 | - | [Liao et al. (2015)](https://doi.org/10.1128/AAC.01343-15) *PS* |
| OMVs pathogenicity (antibiotic resistance) | Ultracentrifugation 150,000 x g, 3 h, 4 °C | ATCC 19606, JU0126 | - | Modified BCA protein assay | [Kesavan, D., Vasudevan, A., Wu, L. et al. (2020)](https://doi.org/10.1186/s12866-020-1722-1) *PSM* |
| OMVs pathogenicity (subproteome analysis) | Ultracentrifugation 200,000 × g, 90 min (2x) | AbH12O-A2 | - | Bradford assay | [Mendez et al. (2012)](http://dx.doi.org/10.1021/pr300496c) *PSM* |
| OMVs pathogenicity (subproteome analysis) | Ultracentrifugation 150,000 x g, 3 h, 4 °C + sucrose density gradient 200,000 x g, 20 h, 4 °C | DU202 | 52 ± 32 | Bradford assay | [Kwon, Sang-Oh et al. (2009)](https://doi.org/10.1111/j.1574-6968.2009.01669.x) *PSM* |
| Vaccination | Ultracentrifugation 150,000 x g, 2 h | ATCC 19606 | < 100 | Micro BCA Protein Assay | [Cai et al., 2019](https://doi.org/10.1155/2019/2835256) |
|  |  | JU0126 | > 100 |  |  |
| Vaccination | Ultracentrifugation 200,000 × g, 90 min, 4 °C | ATCC 19606, Ab-154 and 113-16 | 20 - 60 | - | [McConnell et al. (2011)](https://doi.org/10.1016/j.vaccine.2011.06.001) |
| Vaccination | Ultracentrifugation 200,00 x g, 2 h, 4 °C | Clinical strains | 20 - 100 | - | [Huang W, Yao Y, Long Q, Yang X, Sun W, et al., 2014](https://doi.org/10.1371/journal.pone.0100727) |
| Vaccination | s-OMVs: Ultracentrifugation 12,5000 × g, 2 h, 4 °C + Optiprep density gradient 150,000 × g 5 h, 4 °C | ATCC17978; LAC-4 | 70 - 500 | Lowry method | [Li et al., 2020](https://doi.org/10.3389/fimmu.2020.01069) |
|  | n-OMVs: Centrifugation 5,000 × g, 20 min, 4 °C + centrifugation 30,000 × g, 20 min, 4 °C + ultracentrifugation 150,000 × g, 2 h, 4 °C | ATCC 17978 | 100 - 600 |  |  |
|  | SuOMV:  Ultracentrifugation 360,000 × g, 2 h, 4 °C x2 + sucrose density gradient 250,000 × g, 16 h, 4 °C | ATCC 17978 | 50 - 1000 |  |  |
| Vaccination | Ultracentrifugation 200,000 x g, 90 min, 4°C | ATCC 19606; IB010 (Δ*LpxD*) | 20 - 60 | Bradford assay | [Pulido et al., 2020](https://pdf.sciencedirectassets.com/271205/1-s2.0-S0264410X11X00312/1-s2.0-S0264410X11008619/main.pdf?X-Amz-Security-Token=IQoJb3JpZ2luX2VjEL7%2F%2F%2F%2F%2F%2F%2F%2F%2F%2FwEaCXVzLWVhc3QtMSJHMEUCIQCAvGmocForWQGJlRlLEXE55T9gFLWtCI4UlV%2BcW%2F94RwIgMvY3qxdacRXIbvTqZsJ7Bqy5GzBr00sOiVq3Z9ux1AIqswUIRxAFGgwwNTkwMDM1NDY4NjUiDC%2FxIcVRHBMRN4KW6iqQBaUkgzGgB39X0QHsfjsKKuJ9vDy5suUSGDHU%2FS%2FkS1vm7nNcapABI8R79cmXy1wsVMF3Qmv5grnGHPbyUxScvD6GGkh5dbnTi9whEE76SOggMAF2fse41IRzpm4Jk6oeDDmveW9btY06eWfex8bto6nLlfTLrkoNtAz5Gs4clvJ7l23e4aIDcdL3P6DHJmOCIqZofm74p45tyNhyhUfY8XhNGG41JB%2FYDRJV%2BA3hQ8UmxahD0D7C7HRbSU%2BSFnAW4mzf%2FYSLAgZT0ZXFbPj2Hy7xb7zvWARlA%2FjIC91p%2FjNnUnSSC99nd9alk%2BXFpt9ZxDrf3XLsqIixEaoHwcTS0acjpYtId4fVbnfLqhlWbRnChgo%2FfdYVb81wLNZeUA2lkOXlvaeZ4AfHNOgQ%2FrssUqQhALehPShZopnIeW5mgk%2BAzscEnKV77DcBz9J%2Bhhoy8inY506T3Q00kffbSUU2flWTIoBgf8b7s0p6D7Kjdv6iqer44YEmEJvbUYEAMbrOQzvcKQhBRBe%2B5U3hXEgPryyPWtBnqvL8xjHL6e9IkdEMWxV4m2VdwwjSCEztBgHpnA7IPO3bivekZVmeX56jox4LljQGUN0%2BtkcUUG7WOwEC0dtTJqlDfgzwKt37cfiIAp9wBFaw24R2%2FiwkH6EN1TL6i%2FzdE10%2B4UbuldOwAlXhSCkhbm2YvCOywaSeJITSTiARS%2BbanCp10q17EH2GqETXrNJ%2F9RyOJpd%2FHBPL2z3QsOcI3HaiO45sh5G4FGM7unx2GaumhjQ0CLiMCcxKvMu86bY5PHpE5K9RYN3X83Tmnpuk%2BgDgfWRNFzpAJI3odCGKkTDfsbFB9ugx0ndtyYAY6yRLMLxQRse2GgWgFyTmMO2Ns7kGOrEBTOR6ilQ3AZCOPTzX4hUvrsZikPzCx31UrGJZCzMjQfhJGpUj7r6aSNJdUs3wB4obz9g%2B%2FRzEn0i5IzqgNhz3FbsS0vQLBpP9U3R4DFA%2FkYOFE9CH49WNRguH3tPcND0JDN2WFZXS9W2cwJqdnb8jzrxR3oM3ZeV%2Fe65k8ui1TMAD1PhbE39qSjrM3rIzWhGLtlzGd8aExrwO2ki5NZhQy8dUq5vLptykj0B77SXg9scN&X-Amz-Algorithm=AWS4-HMAC-SHA256&X-Amz-Date=20241107T142924Z&X-Amz-SignedHeaders=host&X-Amz-Expires=300&X-Amz-Credential=ASIAQ3PHCVTYY73FGG42%2F20241107%2Fus-east-1%2Fs3%2Faws4_request&X-Amz-Signature=a8ff28a2548c908482eadd957c9a1ff63ecf780b5a12c5c6bb63d17e1de786ec&hash=59f7384436b07a8c42a686f516db8f028629de9fb7b38c71a2506b9edb7550ca&host=68042c943591013ac2b2430a89b270f6af2c76d8dfd086a07176afe7c76c2c61&pii=S0264410X11008619&tid=spdf-0cb5e19c-ddef-45c8-bf4d-3945a8560abd&sid=0788b57f98930345d12b9dc98dba0f358668gxrqb&type=client&tsoh=d3d3LnNjaWVuY2VkaXJlY3QuY29t&) |
| Vaccination | Ultracentrifugation 200,000 x g, 90 min, 4 °C | WHG40137 | nOMVs 100 -800; LOMVs 50 - 800 nm | BCA protein assay | [Li et al., 2024](https://doi.org/10.1186/s12951-024-02553-x) *PSM* |
| Vaccination | Ultracentrifugation 150,000 g, 2 h, 4°C | Lac-4 | 30 - 5000 | BCA protein assay | [Bjanes et al., 2024](https://doi.org/10.1002/anbr.202200130) |
| Vaccination | Ultracentrifugation, 100,000 g, 2 h, 4 °C | ATCC 17978 | - | Lipophilic dye | [Higham et al., 2023](https://doi.org/10.1016/j.jinf.2023.02.035) |
| Vaccination | Optiprep 100,000 x g, 4°C, 16.30 h + ultracentrifugation 100,000 x g, 2 h, 4 °C | ATCC 19606; IB010 (Δ*LpxD*) | 45 - 500 | Bradford assay | [Cano-Castaño et al., 2024](https://doi.org/10.3390/%20ijms25179272) *PSM* |
|  | Ultracentrifugation 100,000 x g, 6 h, 4 °C x2 | ATCC 19606 | 45 - 600 |  |  |
|  | Ultracentrifugation 100,000 x g, 6 h, 4 °C + SEC columns | ATCC 19606 | 45 - 600 |  |  |
|  | SEC columns | ATCC 19606 | 45 - 600 |  |  |

PS= Publication selected for protein study; PSM= Publication selected for protein study with detailed list of proteins; LB= Luria-Bertani médium; MV= membrane vesicle; PMR= Polimixin B resistant**;** O/N= Overnight; s-OMVs= spontaneously released AbOMVs; n-OMVs= native AbOMVs; SuOMV= sucrose-extracted AbOMV;
